# Reversing the Resistance Phenotype of the *Biomphalaria glabrata* Snail Host *Schistosoma mansoni* Infection by Temperature Modulation

**DOI:** 10.1371/journal.ppat.1002677

**Published:** 2012-04-26

**Authors:** Wannaporn Ittiprasert, Matty Knight

**Affiliations:** Biomedical Research Institute, Rockville, Maryland, United States of America; University of Wisconsin-Madison, United States of America

## Abstract

*Biomphalaria glabrata* snails that display either resistant or susceptible phenotypes to the parasitic trematode, *Schistosoma mansoni* provide an invaluable resource towards elucidating the molecular basis of the snail-host/schistosome relationship. Previously, we showed that induction of stress genes either after heat-shock or parasite infection was a major feature distinguishing juvenile susceptible snails from their resistant counterparts. In order to examine this apparent association between heat stress and snail susceptibility, we investigated the effect of temperature modulation in the resistant snail stock, BS-90. Here, we show that, incubated for up to 4 hrs at 32°C prior to infection, these resistant snails became susceptible to infection, i.e. shedding cercariae at 5 weeks post exposure (PE) while unstressed resistant snails, as expected, remained resistant. This suggests that susceptibility to infection by this resistant snail phenotype is temperature-sensitive (*ts*). Additionally, resistant snails treated with the Hsp 90 specific inhibitor, geldanamycin (GA) after heat stress, were no longer susceptible to infection, retaining their resistant phenotype. Consistently, susceptible snail phenotypes treated with 100 mM GA before parasite exposure also remained uninfected. These results provide direct evidence for the induction of stress genes (heat shock proteins; Hsp 70, Hsp 90 and the reverse transcriptase [RT] domain of the *nimbus* non-LTR retrotransposon) in *B. glabrata* susceptibility to *S. mansoni* infection and characterize the resistant BS-90 snails as a temperature-sensitive phenotype. This study of reversing snail susceptibility phenotypes to *S. mansoni* provides an opportunity to directly track molecular pathway(s) that underlie the *B. glabrata* snail's ability to either sustain or destroy the *S. mansoni* parasite.

## Author Summary

*Biomphalaria glabrata* snails that are either resistant or susceptible to the parasite, *Schistosoma mansoni*, have been an invaluable resource in studies aimed at understanding the molecular basis of the snail/schistosome interaction. Schistosomes cause the chronic debilitating disease schistosomiasis. Thus, it is hoped that dissecting pathways that underlie the snail/schistosome relationship might translate into alternative control strategies that will include blocking transmission of the parasite at the snail-stage of its development. Induction of stress genes is a feature distinguishing early exposed juvenile susceptible NMRI snails from resistant BS-90 snail stocks. To further analyze this apparent involvement of stress induction and snail susceptibility, here we applied heat stress to the resistant BS-90 snail, enhancing induction of stress genes (Hsp 70, Hsp 90 and RT) prior to infection. Results showed these resistant snails became susceptible, indicating resistance as being a temperature sensitive phenotype in these snails. Stressed resistant snails treated with the Hsp 90 specific inhibitor, geldanamycin, prior to exposure, were, however, shown to maintain their refractory phenotype. Interestingly, inhibitor treated susceptible snails also became non-susceptible. Collectively, these data point to stress induction as an important early step in the ability of *S. mansoni* to infect juvenile *B. glabrata* snails.

## Introduction

Schistosomes are parasitic trematodes that cause the chronic debilitating disease schistosomiasis, a neglected tropical disease that persists in over 70 countries of the developing world. It is estimated that at least 200 million people are chronically infected with the parasite with another 800 million remaining at risk for exposure. The disease burden is estimated at over 70 million disability-adjusted life years (DALYs) and there is increasing awareness that schistosomiasis can impact the epidemiology of other infectious diseases such as HIV (especially in female patients with genital schistosomiasis). A concerted effort is, therefore, being made to develop novel intervention tools that include blocking transmission of the parasite at the snail stage of its life cycle [1]–[3].

Freshwater snails serve as obligatory intermediate hosts for the development of parasitic trematodes. Throughout South America and the Caribbean Islands the snail, *Biomphalaria glabrata* plays an important role in the transmission of *Schistosoma mansoni*. The relative ease of maintaining *B. glabrata* in the laboratory has enabled it to become the host/pathogen model system of choice in which studies aimed at elucidating the molecular basis of snail/schistosome interactions are being conducted. Thus far, studies using representative snail stocks that are either resistant or susceptible to the parasite provide an invaluable resource towards unraveling the complex biology of the snail/schistosome encounter. For example, using pedigree snail stocks with varying susceptibility phenotypes, a strong genetic basis was shown to exist for the susceptibility of *B. glabrata* to *S. mansoni* [4]. In adult *B. glabrata*, resistance to *S. mansoni* has been shown to be a dominant single-gene trait that is inherited by simple Mendelian genetics. In juvenile snails, however, genetics of resistance has been shown to be a complex trait, involving 5 to 6 genes each with multiple alleles. Similarly, genetics of susceptibility to the parasite either in juvenile or adult snails has been shown to be multi-genic [5].

Using snail stocks that represent these different susceptibility phenotypes, the genetic locus/loci governing these traits have been assessed by a variety of DNA genotyping tools. These studies have led to the identification of heritable markers that underscore the adult snail parasite resistant phenotype [6]. Advances have also been made towards the identification of genes associated with snail susceptibility phenotypes by examining differences in gene expression profiles between snails that are either resistant or susceptible in response to parasite infection [6]–[10]. Accordingly, several genes involved in the snail's innate defense system are now known to play a significant role in the balance of whether the snail becomes infected or not [11], [12]. For example, in a resistant snail, such as the well-known representative BS-90 stock, the anti-parasite response in this snail has been shown to culminate in the encapsulation of the invading miracidia by a cell-mediated response involving hemocytes that, with plasma (hemolymph) factors, destroys the miracidium within a few days after it penetrates the snail. In a typical susceptible snail, such as the NMRI stock, however, there is no such active innate defense response against the invading miracidium and, therefore, the parasite survives, differentiates into sporocyts, producing cercariae that when released into freshwater can infect a human host, and go on to complete the life cycle.

Aside from the well-recognized genetic basis of the snail-schistosome relationship, shared molecular determinants of both organisms (snail and parasite) are also thought to play a role in the snail host compatibility to *S. mansoni*. Thus, interactions of snail diversified fibrinogen-related proteins (FREPs) and polymorphic mucins of schistosomes have been identified as some of the target molecules of snail and parasite, respectively that either by interacting, or not, with each other define compatibility/incompatibility of the snail/schistosome encounter [13]–[15]. This concept of shared, or molecular mimicry, at the snail - parasite interphase, underlying mechanisms of schistosome-snail compatibility/incompatibility is referred to as the matched- mismatched hypothesis [16].

Variations in susceptibility of *B. glabrata* to *S. mansoni* have been well documented [17], [18]. Furthermore, age- related variations in susceptibility have also been described. For example, Minchella and Richards showed that a snail that is susceptible as a juvenile can become resistant once it reaches adulthood, to the same strain of *S. mansoni* [19]. Given these variations, compounded with the fact that younger snails are, in general, more vulnerable to infection than adults [20], we felt that to identify the mechanism(s) governing susceptibility to *S. mansoni*, in juvenile, rather than adult snails, might be more beneficial in the long run towards our eventual goal of blocking disease transmission in the snail host. For this reason, therefore, the present study was performed entirely with juvenile and not adult snails.

To date, very few studies have investigated the modulation of stress genes and *B. glabrata* susceptibility to *S. mansoni*. However, Lockyer et al. (2004), while examining differential gene expression between resistant and susceptible adult snails, in response to *S. mansoni*, detected upregulation of the transcript encoding the stress response gene, heat shock factor (Hsp) 70 in resistant but not susceptible snails after *S. mansoni* infection [21]. These results are in contrast to those we obtained [22], showing instead upregulation of this transcript in early parasite exposed juvenile susceptible, but not resistant snails. Additionally, unlike the Lockyer et al. study where constitutive expression of Hsp 70 transcript was not observed in either resistant or susceptible adult snails, we showed that the expression of Hsp 70 occurs at similar levels in both normal resistant and susceptible juvenile snails. Furthermore, another study done using hemocytes collected from parasite exposed adult resistant and susceptible snails, showed down regulation of the Hsp 70 protein occurs in hemocytes of both these snails following infection, with more suppression of the transcript in susceptible than in resistant adult snails [23]. Thus, from the above studies, it is clear that there are major discrepancies concerning the expression of Hsp 70 between juvenile and adult resistant and susceptible snails, either with, or without infection, and also from hemocytes removed from infected adult resistant and susceptible snails. These discrepancies notwithstanding, as early as 1954 it was shown that raising the water temperature for maintaining *B. glabrata* shortened the length of the pre-patent period in *S. mansoni* infected snails, and also helped to maintain snail infectivity [24]. Additionally, this early study showed that some snails lost their infections when they were maintained at low temperature. In 1991, Lefcort and Bayne showed that *S. mansoni* infected resistant snails (13–16-R1 stock) displayed a preference for lower temperature compared to similarly exposed susceptible snails. No molecular explanations were, however, provided for these earlier observations [25].

While examining changes in gene expression profiles between juvenile resistant and susceptible snails soon after parasite exposure, we showed that the stress gene, Hsp 70 was induced early in susceptible but not resistant juvenile snails [9], [22]. Subsequently, we showed that the Hsp 70 transcript was co-expressed with the transcript corresponding to the reverse transcriptase (RT) domain of the *B. glabrata* non LTR-retrotransposon, *nimbus*, after exposure of susceptible juvenile snails to normal but not to irradiated miracidia. Similar gene profiling studies done in *B. glabrata* after exposure to another trematode, *Echinostoma paraensi*, also reported the upregulation of Hsp 70 in response to this parasite infection in the snail [26].

Because of this apparent association of an early stress induction and juvenile snail susceptibility, in this study we tested the hypothesis that enhancing stress prior to infection of a representative resistant snail, such as the BS-90 stock, by non-lethal temperature modulation, reverses the resistance phenotype.

The BS-90 snail originally isolated in the 1960s by Paraense and Correa in Salvador (Brazil) is a wild type snail that is resistant at any age (either as juveniles or adults) to both new and old world *S. mansoni* [17]. For this reason, most investigators have, since 1990, used this stock for studies aimed at identifying genes that underlie the aforementioned active innate defense response seen in these snails against *S. mansoni*. At ambient temperature (25°C) susceptible snails, such as the NMRI stock, reliably shed cercariae (varying between 85–95%) within 4 to 6 weeks after miracidia exposure, whereas exposed BS-90 snails destroy the parasite soon after infection, and thereby remain negative. Since arriving in our laboratory in 1990, BS-90 snails have never been known to become susceptible. Furthermore, in an early series of experiments conducted by Paraense and Correa (1963) where these snails were exposed to *S. mansoni* under different temperature conditions during the coldest (19.5–22.7°C) and warmest (24.9–27.6°C) months in the laboratory, no effect of temperature was detected. Indeed, in this same study, the snails derived from the original stock (collected in a lake near a beach at Amaralina district in Salvador), exposed to up to 100 miracidia remained negative.

Here, we show that by subjecting the resistant BS-90 stock to non-lethal heat shock treatment at 32°C, herein referred to as heat-pulse, prior to exposure, the snails consistently reversed their phenotype, shedding cercariae 5 weeks after infection. In contrast, similarly exposed, but unstressed BS-90 snails, remained uninfected. Additionally, if the stressed BS-90 snails were immediately treated with the Hsp 90 inhibitor, geldanamycin (GA) before exposure, they remained resistant. Interestingly, treatment of the highly susceptible NMRI snails with 100 mM of the same inhibitor before exposure to *S. mansoni* also prevented infection. These findings are consistent with an apparent association of the induction of stress genes (Hsp 70, Hsp 90 and RT) and *B. glabrata* susceptibility to *S. mansoni*.

Furthermore, the temperature sensitive switching of the resistant phenotype of *B. glabrata* to *S. mansoni* susceptibility provides an important means of directly tracking mechanisms that underscore the parasite's survival or destruction in the *B. glabrata* intermediate snail host.

## Materials and Methods

### Ethics statement

Female SW mice were purchased from Taconic (Germantown, NY) and maintained in the Biomedical Research Institute's (BRI) animal facility, which is accredited by Lewis et al. [27], [28] the American Association for Accreditation of Laboratory Animal Care (AAALAC; #000779), is a USDA registered animal facility (51-R-0050), and has an Animal Welfare Assurance on file with the National Institutes of Health, Office of Laboratory Animal Welfare (OLAW), A3080-01. Maintenance of the mice, exposure to *S. mansoni* cercariae, and subsequent harvesting of the adult worms were approved by the BRI Institutional Animal Care and Use Committee (IACUC protocol approval number 09-03). All procedures employed were consistent with the Guide for the Care and Use of Laboratory Animals.

#### Snails

Juvenile *B. glabrata* snails (4–6 mm in diameter) were used throughout the study. The aforementioned resistant, BS-90 (0% infectivity rate to *S. mansoni*) at ambient temperature [17] and susceptible, NMRI snail stocks (85–95% susceptibility rate to *S. mansoni*) [29] were employed, and the NMRI [30] strain of *S. mansoni* was utilized for all snail exposures. The snails were maintained in aerated water (∼23–25°C) and fed romaine lettuce. Snails were placed in sterile distilled H_2_O overnight before all experiments were conducted.

### Heat-pulse, snail exposure and cercarial shedding

Juvenile snails were subjected to heat-pulse by incubation in pre-warmed (32°C) sterile water for 1–4 hrs as previously described [22]. After the heat-pulse treatment, the snails were immediately exposed to *S. mansoni* miracidia (10 miracidia/snail) at ambient temperature (25°C) in fresh aerated tap water for at least 3 hrs, individually, as previously described [9]. The heat-pulse treatment of juvenile resistant snails was performed on the basis of our previous data that showed optimal induction of Hsp 70 and RT transcripts occurred in these snails only after prolonged (2 to 4 hrs) heat stress [22]. Infected snails were maintained as described above, but in the dark. After 4 weeks, snails were screened individually for cercarial shedding by placing each snail in the well of a 12 well -plate (∼3 ml aerated water in each well) under a light source at room temperature for 1 hr as previously described [22], [31]. Given that the objective of this study was to examine the effect of temperature modulation on juvenile resistant snails, we scored snails as susceptible if they released any cercariae at all after infection, and not by how many parasites were shed per snail. This method of scoring was chosen to reflect the real life situation that it takes only a few viable parasites to transmit schistosomiasis in endemic regions. The water in individual wells was subsequently examined for the presence, or absence of cercariae under a dissecting microscope. The snails that were not shedding cercariae at the 9^th^ week post-exposure, however, were kept and examined every week until the 12^th^ week thereafter the snails were monitored on a monthly basis for cercarial shedding.

### Geldanamycin, Hsp 90 inhibitor treatment

After heat-pulse treatment as described above, BS-90 snails were transferred immediately into a beaker containing a solution of 100 mM geldanamycin (GA) (Sigma Aldrich) overnight at room temperature. Snails were removed from the drug solution, washed twice (5 min intervals) in ∼30 ml of fresh aerated water at room temperature to rinse off residual contaminating drug before exposing to miracidia individually in 2 ml of fresh aerated water (in a 12 well plate). Each snail was exposed in fresh water at room temperature while monitoring under a dissecting microscope for miracidia penetration. All snails were exposed to twice the number of miracidia (10 miracidia/snail) we normally use for exposing juvenile NMRI snails.

Different types of experimental conditions (12 snails for each cohort) were set-up as follows: 1) normal (unstressed and unexposed) BS-90 snails, 2) parasite exposed-normal BS-90 snails, 3) heat-pulsed only (unexposed) BS-90 snails, 4) exposed then heat-pulsed BS-90 snails, 5) heat-pulsed/GA treated-exposed BS-90 snails, 6) GA treated normal BS-90 snails. Water changes were done on a weekly basis on all the snails. Snails were examined for evidence of parasite infection (cercarial shedding) as described above. In this study, aerated fresh water collected from the same container (30 gallons capacity barrel) was used for all snail husbandry and miracidia exposures.

To examine the effect of GA treatment on *B. glabrata* NMRI susceptible snails and parasite exposure, juvenile snails (4–6 mm in diameter) were used for the study. Twelve snails in a cohort were treated with GA at final concentrations, 0, 0.1, 1.0, 10 and 100 mM. Snails were treated as described above by incubating overnight in a 50 ml beaker containing the drug solution. Treated snails were removed from the drug solution, washed as described above before being transferred into 2 ml of aerated fresh water (in a 12 well plate) for individual miracidia exposure. Each snail was exposed in freshwater at room temperature (10 miracidia/snail) and monitored under a dissecting microscope for miracidia penetration. After exposure, snails were transferred into fresh water for the remainder of the study. Experiments were repeated, 5 times, for the highest dose (100 mM) of GA, and 3 times for the lower drug concentrations, representing 5 and 3 biological replicates, respectively. Twelve size-matched snails were used for each drug concentration. As a control, miracidia were treated with the same high dose of GA for 5 hrs then used for snail exposures as described above. Data were pooled (12 snails/dose of drug) from 5 independent experiments for the high (100 mM) concentration (N = 60) and 3 independent experiments from the low (0.1–10 mM) concentration (N = 36) and standard error (SE) determined. In addition, three other controls (12 snails for each cohort) were used as follows: 1) normal NMRI snails 2) exposed normal NMRI snails and 3) unexposed GA treated-NMRI snails. Water changes were done on a weekly basis on all the snails, and exposed snails were examined for evidence of parasite infection (cercarial shedding) as described above. From the 3 and 5 biological replicates (N = 36, and N = 60, respectively), one-way ANOVA was used to determine if differences in cercarial shedding (%) between low and high doses of GA treatment of NMRI snails were significant.

### RNA isolation, real time quantitative and qualitative RT-PCR analysis

To investigate the expression of stress genes Hsp 70, Hsp 90 and RT, snails from the same cohort, subjected to heat-pulse, and exposed individually to miracidia (10 miracidia/snail) (as described above) were snap frozen in liquid nitrogen and kept at −70°C until required for RNA isolation. Of the three cellular stress genes examined in the present study, the differential induction of Hsp 70 and RT between susceptible NMRI and resistant BS-90 snails after parasite exposure have previously been reported [22]. To determine differences in the expression of Hsp 90 between juvenile BS-90 and NMRI snails they were exposed, individually as described above for 0, 15, 30, 45, 60 and 120 min before being frozen for RNA isolation.

Total RNA was extracted from the whole snail by single-step simultaneous RNA isolation using RNAzol RT (Molecular Research Center, Inc., OH) [32]. RNA from individual snails was utilized for all qPCR analysis. The quantity and quality of RNA was determined by UV absorbance (A260) and an A260/A280 ratio (∼1.9–2.0) obtained by using the NanoDrop 1000 (Thermo Scientific). Eighty nanograms of total RNA was analyzed by real time qPCR using Brilliant II SYBR Green QRT-PCR Master mix according to the manufacturer's instructions (Stratagene, CA). Real time qPCR reactions were done using the 7300 real - time PCR system from ABI (Applied Biosystem). At the beginning of the assay, a validation protocol (done according to the manufacturer's instructions) was performed to test amplification efficiencies of stress genes (Hsp 70, Hsp 90 and RT) and myoglobin (constitutively expressed house keeping gene) [9], [22], [33], [34], [35]. The validation experiment was done using four different RNA templates dilutions to confirm that amplification efficiencies were equal between our genes of interest and myoglobin genes (data not shown). The 25 µl final reaction volume contained either 200 nM of gene specific primers for Hsp 70, Hsp 90 and RT or 50 nM of primers for the house keeping myoglobin gene that amplifies a 349 bp fragment [33]. Nucleotide sequences of the gene specific primers for Hsp 70 and RT have previously been described [22]. The Hsp 90 specific primer pair (forward primer; 5′-tgtgcgcagagtgttcatcatgg-3′ and reverse primer; 5′-ctcctgtgaggcttcaatgagtc-3′) was designed by Primer-Blast software (http://www.ncbi.nlm.nih.gov/tools/primer-blast/index.cgi?LINK_LOC=BlastHome) from publically available Expressed Sequence Tags (ESTs of *B. glabrata* 5′end clone; accession number, AA547771 that has homology to heat shock protein 90). The Hsp90 gene specific primers amplify a 199 bp fragment from *B. glabrata* BS-90 and NMRI stocks. All primers were checked for non-cross amplification with *S. mansoni* cDNA template as well as with other schistosome parasite species (data not shown). Reactions were performed in triplicate (technical replicates) for each individual snail RNA sample in a one-step format with the first strand cDNA synthesis and real time PCR amplification done as described previously [35]. Each reaction contained a no template negative control to rule out non-specific amplification from contamination in the primers and buffers. The gene transcript levels were normalized relative to myoglobin expression. In our study, myoglobin showed stable expression in BS-90 and NMRI snails under normal and stress conditions (data not shown). After five biological replicates using 10 snails/time point (50 snails in total) the fold change in transcription of corresponding genes of interest were calculated by using the formula below [36].
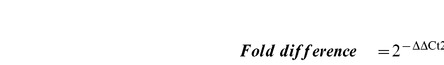



*P*-values were calculated by comparing the delta-Ct value as previously described for each group by Student's *t*-test to determine if the differential expression of the transcripts between experimental and control groups was significant (9, 22, 32, 33). Fold-changes in transcripts corresponding to stress genes of interest, normalized relative to myoglobin expression from individual snail RNA (10 snails/time point, assayed in triplicate), were determined from five independent experiments. These data were pooled (N = 50) and standard error (SE) determined.

Each reaction contained a no template negative control to rule out non-specific amplification from contamination in the primers and buffers. To monitor the differential transcription of Hsp 90 between NMRI and BS-90 snails, RNAs were isolated at different time points following exposure, as described above, and were normalized relative to myoglobin expression.

To determine constitutive expression of Hsp 90 in both BS-90 and NMRI snails, we performed qualitative RT-PCR as previously described [35] using first strand cDNA synthesized from total RNA using a cDNA synthesis kit (Promega) with M-MLV reverse transcriptase isolated from two same-sized (4–6 mm in diameter) individual snails of either the resistant (BS-90) or the susceptible (NMRI) stock. Using the Hsp 90 gene specific primers described above, second strand PCR was performed with cDNA template that was prepared either in the presence or absence of M-MLV RT under the following conditions: denaturation step at 95°C for 30 sec, annealing step at 65°C for 30 sec, extension at 72°C for 1 min, repeated for 29 cycles. The PCR products were resolved by agarose gel electrophoresis (1.2%) and amplicons were visualized by ethidium bromide staining under UV trans-illumination using the Quantity One software (Gel Doc XR imaging system, BioRad). A 100 bp ladder from a commercial source (Invitrogen Laboratories, CA) was utilized as standard for the qualitative analysis. Parallel PCR-amplification using equal amounts of the same cDNA template was done by using gene specific primers corresponding to the house keeping myoglobin gene as previously described [33]. PCR amplifications were also done without cDNA template, as negative control, to monitor the reagents utilized in the assay for possible contamination.

### Mouse infections and harvesting of *S. mansoni* adult worms

Snails were screened as described above for cercarial shedding and those found to be positive at 7 weeks PE were allowed to shed into an appropriate container for 30–60 min under a light source at room temperature. After shedding, the snails were removed from the container and the cercariae suspension was allowed to stand (10 min at room temperature) to allow any non-specific sediment (mucoid material and feces) from the snail to settle at the bottom of the container. The sediment-free top part of the cercariae suspension was transferred to another container and was used for the mouse infection.

Two female Swiss-Webster (SW) mice (2–3 months old weighing18 grams) were used for the cercariae infection (50 cercariae/mouse) by tail exposure, allowing the cercariae to penetrate individually for ∼30 min. Forty-nine days after infection, feces from the mice were examined for first appearance of parasite eggs. The positive mice were euthanized for recovery of adult worms by the perfusion technique using 0.1 M citrate-0.15 M sodium chloride solution [28].

### Isolation of adult worm DNA and RAPD-PCR genotyping

Mice infected with cercariae that were shed from the ‘resistant’ heat- pulsed -exposed BS-90 snails were perfused at 6 weeks post infection as described by Lewis et al. [28]. Harvested adult worms were frozen individually at −70°C until required. Genomic DNA was isolated from frozen individual worms as described by Simpson et al. [37], and screened by RAPD-PCR using random primers OPR-14 and OPA-O6 (EurofinsMWG/Operon, AL) as previously described [6]. Amplified fragments were separated by agarose gel electrophoresis, and the presence/absence of bands visualized by ethidium bromide staining under UV Trans-illumination (ChemiDoc imaging system, BioRad CA). For comparison, an equal amount of DNA from adult *S. mansoni* harvested from the susceptible snail was analyzed in parallel, using the same random primers mentioned above.

## Results

### Constitutive expression of Hsp 90 in BS-90 and NMRI snails is similar

**Figure 1:**
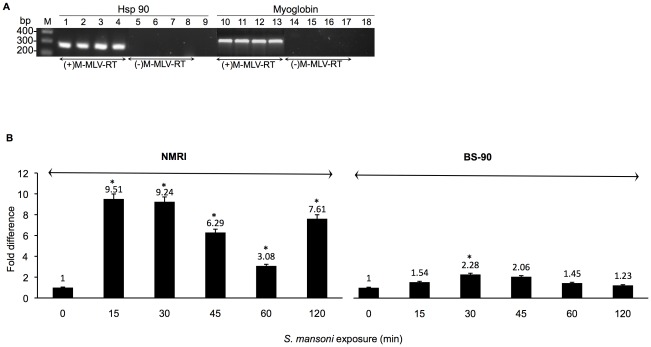
Expression of the Hsp 90 transcript in resistant BS-90, and susceptible NMRI juvenile snails, without and with parasite exposure. (**A**) Constitutive expression of Hsp 90 and myoglobin in two juvenile BS-90 (lanes 1, 2, 5, 6, 10, 11, 14 and 15) and NMRI (lanes 3, 4, 7, 8, 12, 13, 16 and 17) snails by qualitative RT-PCR, using cDNA synthesized with [+M-MLV-RT], or without [−M-MLV-RT] RT in the first strand reaction. The same cDNA template was utilized for PCR amplification but with gene specific primers (forward and reverse) corresponding to the specific transcripts. Amplified bands of the expected size, 199 bp for Hsp 90, and 350 bp for myoglobin stained by ethidium bromide after agarose gel electrophoresis are shown. PCR amplification conducted without cDNA template is shown in lanes 9 and 18. The 100 bp ladder run in parallel is shown on the left. (**B**) Analysis of the fold difference in Hsp 90 expression by real time qPCR between NMRI and BS-90 snails either without (0) *S. mansoni* miracidia exposure or after different time points (15 to 120 mins) PE. The histograms in 1B represent pooled data from 5 independent experiments, each performed with RNA (assayed in triplicate) isolated from 10 individual size-matched juvenile snails. The asterisk * indicates statistical significant *P- values* of <0.05 by using ANOVA.

To investigate if the expression of Hsp 90 in the resistant (BS-90) and susceptible (NMRI) snail would be similar as was shown previously for the levels of Hsp 70 and the *nimbus* RT domain, qualitative RT-PCR was conducted with template cDNA prepared with and without M-MLV-RT from RNA isolated from uninfected individual snails. As shown in Figure 1A, qualitative RT-PCR revealed that basal constitutive expression of Hsp 90 in both snail stocks was remarkably similar. Thus, the PCR amplification of cDNA from two individual BS-90 snails (Fig. 1A, lanes 1 and 2), using the Hsp 90 gene specific primers described in Materials and Methods, produced a 199 bp expected-size band that was similar in size and intensity (Fig. 1A, lanes 1 to 4) to the PCR amplification product produced from cDNA templates from two individual NMRI susceptible snails (Fig. 1A, lanes 3 and 4). To rule out any possibility that the amplicons detected in lanes 1 to 4 might have originated from genomic DNA contamination in RNA preparations utilized for first strand cDNA synthesis, control reactions performed without reverse transcriptase (−M-MLVRT) in the first stand reaction were also utilized for PCR amplifications (Fig. 1A, lanes 5 to 8). Thus, using the Hsp 90 gene-specific primers for amplification reactions with this control (minus M-MLV RT) template produced no bands in either the BS-90 (Lanes 5 and 6) or NMRI (Lanes 6 and 7) samples, indicating that there was no genomic DNA contamination in the RNA preparations utilized for this assay. In parallel RT-PCR was performed with primers corresponding to the housekeeping myoglobin gene using equal amounts of the cDNA templates utilized in lanes 1 to 4. In this case, (also in lanes 10 to 13) an expected 350 bp size PCR product was obtained, indicating similar constitutive expression of myoglobin in both these two snail stocks. As another negative control, PCR was also conducted without cDNA template. The absence of a product (lanes 9 and 18) showed there was no contamination in the reagents utilized for the assay.

### Early induction of the Hsp 90 transcript occurs in susceptible but not in resistant juvenile snails exposed *to S. mansoni*

Since previous studies showed dramatic differences in the temporal modulation and degree of induction of the Hsp 70 and RT transcripts following *S. mansoni* infection between resistant and susceptible snails, in the present study we chose to examine whether parasite exposure mediates the differential expression of another major cellular stress gene, Hsp 90. The induction of the Hsp 90 transcript in susceptible NMRI and resistant BS-90 juvenile snails exposed for different time points (0, 15, 30, 45, 60 and 120 min) to miracidia is shown in Figure 1B. RNA isolated from the snails was analyzed by real time qPCR as described in Materials and Methods using the uniform constitutive expression of the myoglobin transcript in both snail stocks as internal standard. From 5 independent (biological replicates) assays done by using 10 individual snails per time point, with each RNA sample run in triplicate, results showed that as early as 15 min post- exposure almost 10-fold induction of the Hsp 90 transcript was obtained in the susceptible NMRI snail. Furthermore, the transcript remained upregulated in the susceptible snail throughout the 120 min PE time period examined. In contrast, a smaller difference in the upregulation of this transcript (1.54 fold change) occurred in the resistant snail at the early 15 min time point after infection. Although variations in the induction level (of Hsp 90 transcript) were observed in the infected susceptible snail between the early 15–120 min PE time period, none of the levels we detected in the infected resistant snail (1.45 to 1.54 fold change) exceeded those detected in the infected susceptible snail.

### Heat-pulse followed by *S. mansoni* exposure, enhances induction of stress response transcripts in resistant BS-90 snails

**Figure 2:**
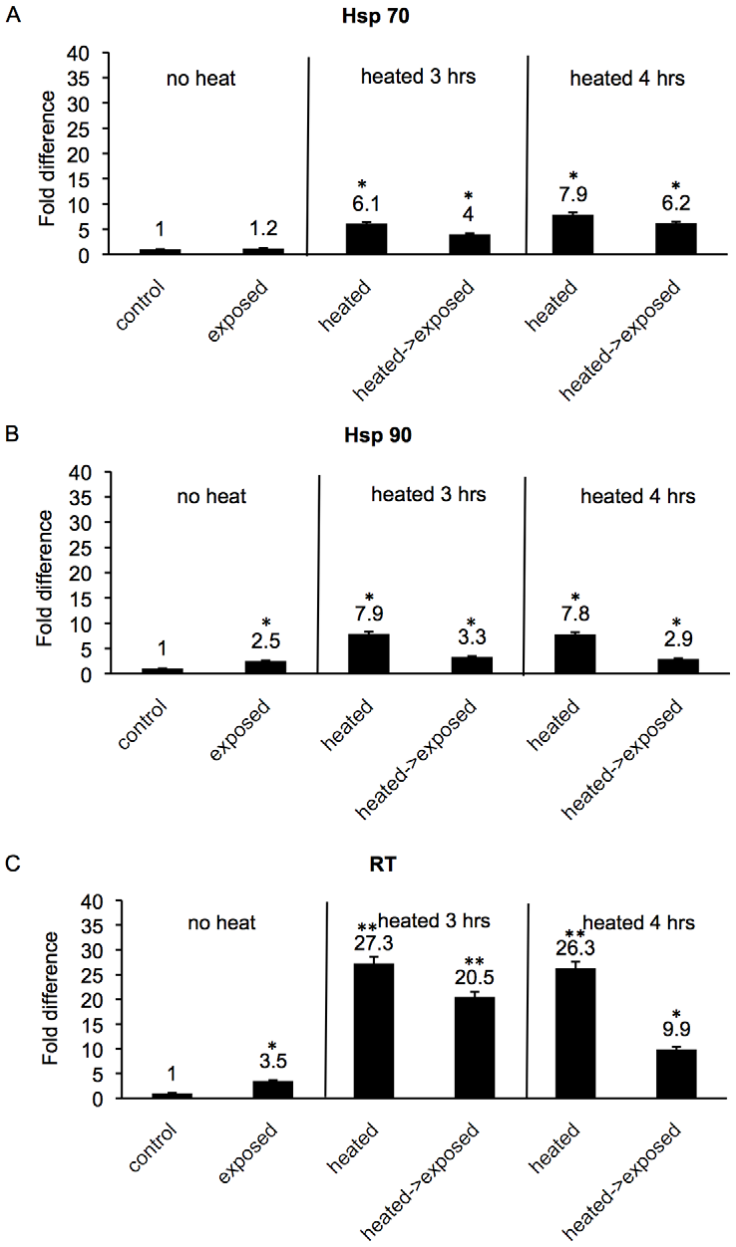
The differential expression of transcripts encoding Hsp 70, Hsp 90 and RT in parasite - exposed, heat-pulsed -parasite- exposed BS-90 juvenile snails. Real time qPCR examination of fold differences in expression of Hsp 70 (panel A), Hsp 90 (panel B), and RT (panel C) in normal BS-90 (control), *S. mansoni* exposed, heat-pulsed (32°C), and heat-pulsed combined with immediate parasite exposure. All exposures were done for 2 hrs using snails that were either not subjected to heat-pulse (no heat), or kept for 3 and 4 hrs at 32°C prior to exposure. The histograms represent pooled data from 5 independent experiments, each performed with RNA (assayed in triplicate) isolated from 10 individual size-matched juvenile snails. The asterisk * and ** indicates statistical significant *P-values* of <0.05 and <0.01, respectively analyzed by Student *t*-test (N = 50).

Previously we ascertained, as mentioned above, that induction of stress genes occurs in the resistant BS-90 snail after a long 2–4 hrs time period either in response to parasite exposure, or heat shock. Accordingly, here we kept the BS-90 snails at 32°C for 3–4 hrs before exposing them immediately to miracidia as described in Materials and Methods. Using real time qPCR, modulations in expression of the three cellular stress genes (Hsp 70, Hsp 90 and RT) were assessed. The fold changes in expression of transcripts corresponding to Hsp 70 (Fig. 2a), Hsp 90 (Fig. 2b) and RT (Fig. 2c) in resistant BS-90 juvenile snails either following heat-pulse treatment, or subsequently subjecting the heat- pulsed snails to *S. mansoni* exposure for 2 hrs were determined (Fig. 2). As shown in Figure 2a, in comparison to snails exposed without prior heat-pulse treatment, minor induction (1.2 fold) of the Hsp 70 transcript was detected in these resistant snails after parasite exposure. In contrast, however, the heat-pulse for either 3 or 4 hrs promoted a more significant induction (6.1 and 7.9 fold, respectively) of this transcript. Interestingly, exposing the snails to miracidia immediately after 3 and 4 hrs heat-pulse kept the induced Hsp 70 transcript at a level that was higher (4 and 6.2 fold induction, respectively) than the 1.2 fold induction we observed in snails that were exposed without being heat pulsed.

In Figure 2b, while a 2.5 fold induction of the Hsp 90 transcript was observed in the unstressed 2 hrs parasite-exposed BS-90 snail, we detected a 7.9 and 7.8 fold increase in this transcript in snails subjected to 3 and 4 hrs of heat-pulse, respectively. The fold change in the Hsp-90 transcript remained relatively higher in snails that were heat-pulsed and immediately exposed to miracidia (3.3 and 2.9 fold increase) compared to the 2.5 fold increase observed in the normal (minus heat-pulse) parasite-exposed snail.

In Figure 2c, in unstressed BS-90 snails exposed to miracidia, a 3.5 fold increase in the RT transcript was observed compared to a 27.3 and 26.3 fold induction of this transcript when snails were kept for 3 and 4 hrs at 32°C prior to infection. Thus, in this case, the dramatic increase in the RT transcript remained elevated in snails that were heat -pulsed for 3 to 4 hrs and then immediately exposed to miracidia. Thus, a strong 20.5 and almost 10 fold increase was detected in these snails (heat-pulse plus exposure) compared to snails that were exposed without heat-pulse treatment where only a 3.3 fold increase in the RT transcript was observed.

The above results showed that compared to snails that were only exposed to miracidia, the induction of all the stress transcripts examined (Hsp70, Hsp90 and RT) was more elevated in BS-90 snails that were responding to parasite exposure after heat - pulse treatment.

### Heat-pulse immediately preceding parasite exposure of resistant snails makes them susceptible

**Figure 3:**
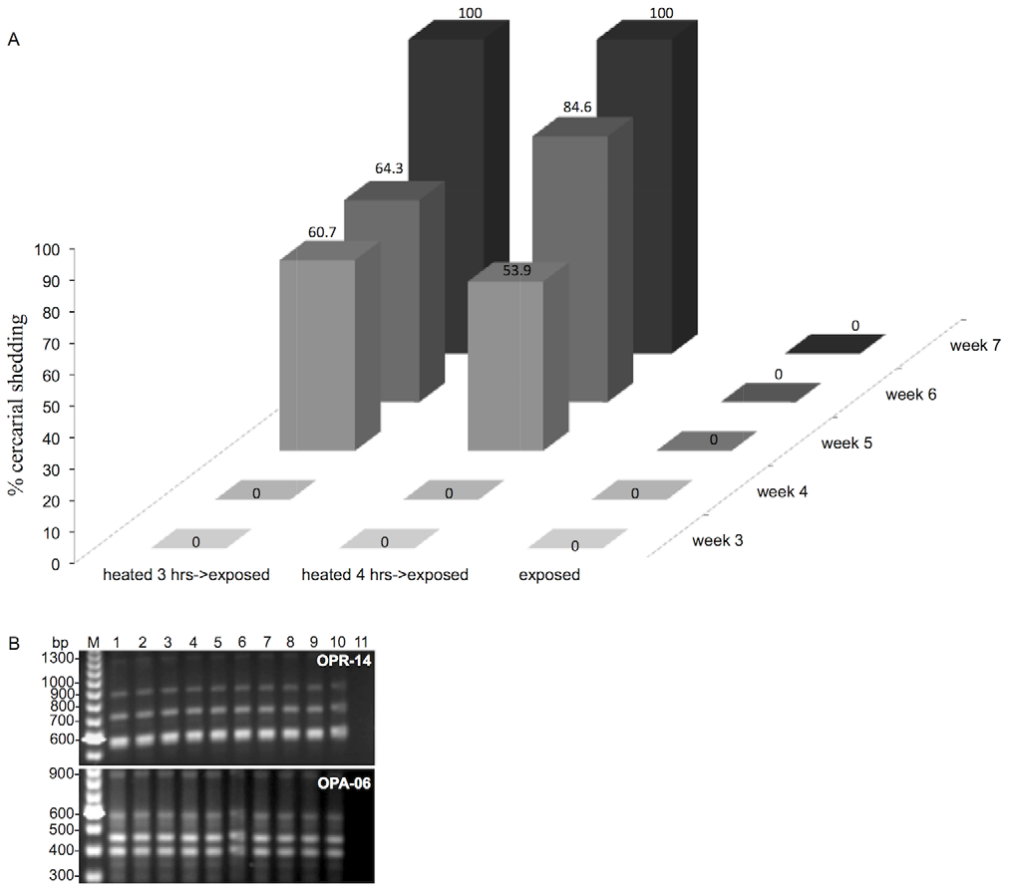
Genotyping of adult *S. mansoni* parasites generated from cercariae released from resistant BS-90 snails following the combination of heat-pulse and parasite infection. (**A**) Percentage of BS-90 resistant snails shedding cercariae after 7 weeks parasite exposure. Note that snails that were exposed to *S. mansoni* without being subjected to the heat-pulse treatment, as expected, remained negative (0) whilst those kept for either 3 hrs or 4 hrs prior to exposure began shedding cercariae at 5 weeks PE. (**B**) RAPD-PCR genotyping of the genomic DNA from individual (lanes 1–7) male worms harvested from mice infected with cercariae released from BS-90 ‘resistant’ snails. Note that the DNA profiles of these parasites, generated with two different random primers, OPR-14 and OPA-06, were invariant when compared to profiles generated with the same primers but with DNA from individual (lanes 8–10) worms harvested from mice that were infected with cercariae shed from susceptible (NMRI) snails. The negative control, i.e. amplification without DNA template is shown in lane 11 and the lane labeled M shows position of 100 bp ladder, run in parallel as standard.

Results in Figure 3a shows the effect of combining heat -pulse treatment with immediate exposure of the stressed resistant snails to miracidia. Parasite-exposed unstressed snails were monitored as control. As shown in Figure 3a, as expected, juvenile resistant snails that were exposed to miracidia without heat -pulse treatment released no cercariae for the entire 7 weeks duration of the experiment. In contrast, a significant number, more than half (60.7% and 53.9%), of resistant snails that were heat -pulsed either for 3 or 4 hrs prior to exposure were found to shed cercariae starting at 5 weeks PE. Interestingly, by 7 weeks PE, all such prior heat -pulsed, and parasite -exposed resistant snails were shedding cercariae.

To determine if cercariae released from these positive resistant snails were biologically viable i.e. capable of infecting the experimental mouse host and developing successfully into adult worms in this host, cercariae released from ‘resistant’ snails were utilized for mouse infections as described in Materials and Methods. Since we were able to perfuse adult worms from the mice infected with ‘resistant’-snail derived parasites, we can conclude that larval parasites released from these ‘resistant’ snails were indeed infectious, developing normally within the expected 6 weeks time-frame into adult worms in the infected mouse. To determine whether these adult worms were in fact genotypically identical to parasites harvested from mice exposed to cercariae released from our representative susceptible snails (NMRI stock), we analyzed genomic DNA from worms harvested from mice that were infected by using either resistant, or susceptible snail derived parasites, with the multi-locus RAPD-PCR genotyping tool. As shown in Figure 3b, the DNA profile, using random primer OPR-14, from 7 individual worms (lanes 1 to 7) was comparable to that of an adult worm recovered from a mouse (by perfusion) that was exposed to cercariae shed from the representative susceptible snail (lanes 8 to 10). Likewise, DNA profiling of the same samples as utilized in lanes 1 and 7 but amplified, this time, with random primer OPA-06 showed no polymorphisms existed between either resistant (lanes 1 to 7) or susceptible (lanes 8 to 10) snail derived parasites. The absence of bands in lane 11, using either of the two random primers but without template DNA shows there was no contamination in either the buffer or enzyme employed for the RAPD assay.

### Miracidia exposure before heat-pulse maintains the refractory phenotype of the resistant snail

**Figure 4:**
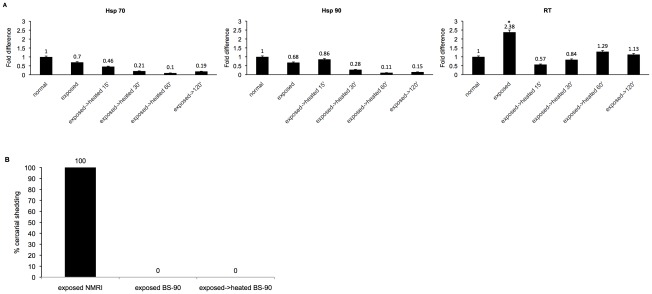
Expression of stress –related transcripts in parasite exposed juvenile resistant BS-90 snails followed by heat-pulse treatment for various time points (0–120 min). (**A**) Real time qPCR analysis of the fold difference in expression of transcripts corresponding to Hsp 70, Hsp 90, and RT in either normal BS-90 resistant snails, exposed snails, or those that were first exposed to *S. mansoni* before being subjected to heat-pulse at 32°C for various time points (15–120 mins). These data were pooled from 10 biological replicates of 5 independent experiments. Note the lack of up-regulation of all transcripts in snails that were exposed and then subsequently kept at 32°C. The asterisk* denotes significant upregulation of one of the transcripts, RT, by Student *t*-test with *P-value*<0.05 (N = 50). (**B**) Results showing 100% shedding of cercariae from susceptible NMRI snails after 7 weeks PE to *S. mansoni* miracidia compared to similarly exposed normal BS-90 snails, or those that were exposed before being subjected to heat pulse treatment at 32°C. It is clear that no cercariae were shed from BS-90 snails in either of these two groups. These data were from 5 biological replicates.

As shown above, heat -pulse treatment followed immediately by miracidia exposure of the resistant snail, resulted in the robust resistant BS-90 snail becoming susceptible. On the basis of the above results, we, therefore, felt it was necessary to determine the outcome of exposing the resistant snails to miracidia before subjecting them to the heat -pulse treatment. Thus, juvenile BS-90 snails were initially exposed to miracidia, and immediately heat -pulsed at 32°C for 3 hrs. Figure 4a shows the real time qPCR results of the fold difference in expression of transcripts encoding Hsp 70, Hsp 90 and RT at different time points (0, 15, 30, 60 and 120 min) after maintaining the infected snails at 32°C. Interestingly, unlike results shown in Figure 2 where enhanced induction of all three transcripts was detected in snails that were heat pulsed before exposure, in this experiment, induction of all the stress transcripts examined remained relatively unchanged.

Accordingly, we monitored these snails (parasite- exposed then heat -pulsed) for cercarial shedding as described above. Results (Fig. 4b) showed that all (100%) the resistant snails that were exposed to the parasite before being subjected to heat -pulse treatment remained negative. From these results, it is clear that exposing these resistant snails to the parasite before they are stressed, maintains the refractory status of these snails, further demonstrating the temperature sensitivity of BS-90 resistant phenotype to *S. mansoni* infection.

### Treatment of heat -pulsed and *S. mansoni* exposed BS-90 snails with the Hsp 90 inhibitor, geldanamycin (GA) blocks susceptibility to infection

**Figure 5:**
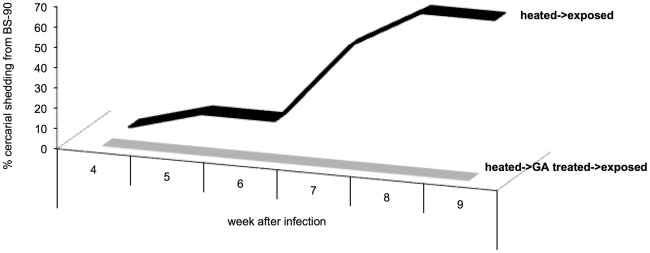
Percentage cercarial shedding from heat-pulsed resistant BS-90 snails that were either treated (+GA), or untreated (−GA) prior to *S. mansoni* exposure. Note that the snails that were not treated with GA but heat- pulsed and exposed began to shed cercariae at 4 weeks PE (with 70% shedding parasites at 9 weeks PE). In contrast, note that the heat -pulsed- GA-treated - infected snails remained negative for the entire 9 weeks duration of the experiment.

To determine, more precisely, if significant upregulation of stress -related transcripts is indeed a factor in rendering the heat -pulsed resistant snails susceptible to infection, we investigated the effect of blocking the action of one of the stress proteins, Hsp 90, by using a specific inhibitor drug geldanamycin (GA) that prevents this protein from performing its role as an essential chaperone in the cell [38]–[40]. In Figure 5, resistant snails that were heat -pulsed for 3 hrs were exposed either immediately to miracidia as described above, or were treated with GA (100 mM) before being exposed to miracidia. Results showed that without inhibitor treatment of stressed snails, the majority (70%) of heat -pulsed and exposed snails were found to shed cercariae 9 weeks after exposure. In contrast, snails that were heat –pulsed, and immediately treated with GA before exposure failed to shed cercariae, remaining negative for the entire 9 weeks duration of the experiment. These data clearly show that blocking the action of Hsp 90 by GA treatment in the stressed resistant snails before exposure maintained their refractory phenotype, thereby indicating that a sustained significant stress induction of Hsp 90 was involved in the mechanism(s) of *B. glabrata* susceptibility to *S. mansoni*.

### Treatment of susceptible NMRI snails with GA before parasite infection renders them non-susceptible to *S. mansoni*

**Figure 6:**
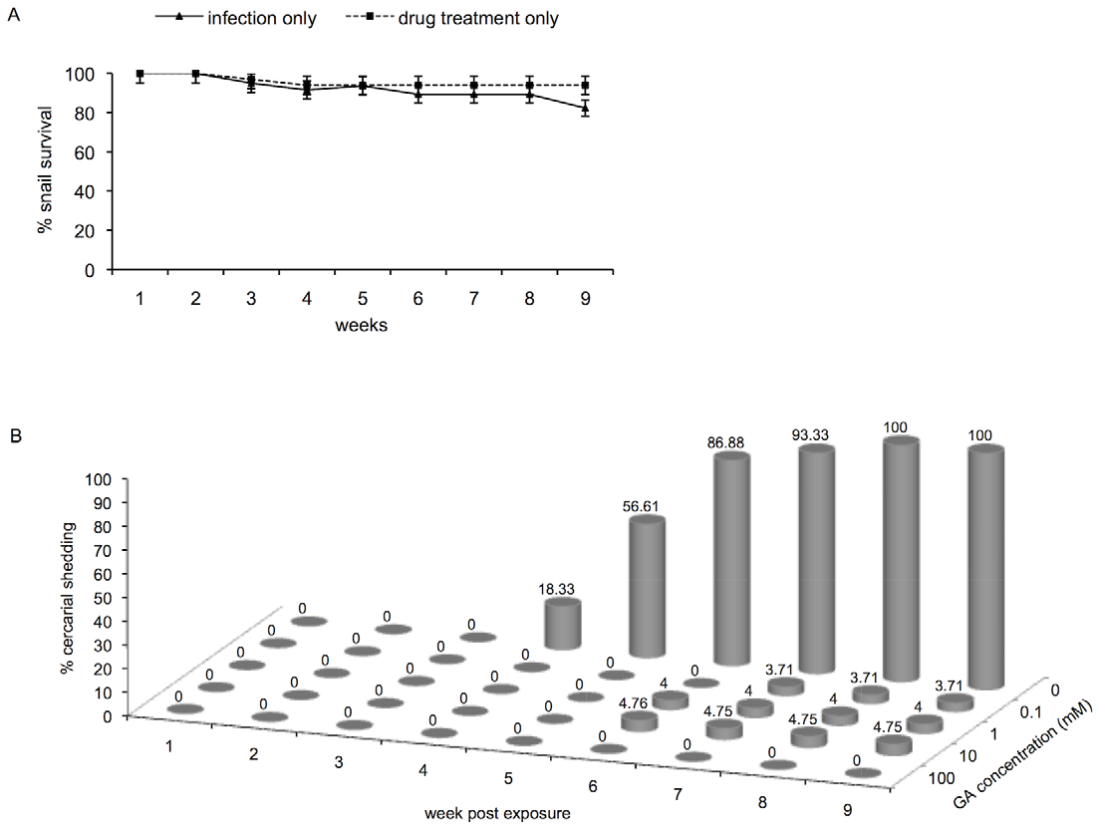
Survival, and cercarial shedding from juvenile susceptible NMRI snails after GA treatment. **A**) The percentage of susceptible NMRI snails surviving after either 9 weeks PE to *S. mansoni* or after treatment with 100 mM GA as described in Materials and Methods. Note that the inhibitor had no toxic effect on the snails. (**B**) Percentage cercarial shedding from GA treated and untreated susceptible (NMRI) snails weeks after infection to *S. mansoni*. Note that at 5 weeks PE, more than 50% of untreated (0) snails were shedding cercariae while all GA treated snails, even those treated with low doses (0.1–10 mM), remained negative for cercarial release. Data were pooled (12 snails/dose of drug) from 5 independent experiments for the high (100 mM) concentration (N = 60) and 3 independent experiments from the low (0.1–10 mM) concentration (N = 36) for standard error (SE) determination. At 9 weeks PE all snails treated prior to exposure with the higher dose of inhibitor remained negative for infection.

To further demonstrate the link between stress induction in juvenile *B. glabrata* snails and their susceptibility to *S. mansoni*, here the effect of pre -treating the susceptible NMRI snail with the aforementioned Hsp 90 inhibitor (GA) on the outcome of infection was examined. Figure 6a shows the survival of snails after either infection alone (minus GA inhibitor) or after treatment with 100 mM of GA. Results (Fig. 6a) showed that snails tolerated the drug at this dose and survived at the same rate (100%) as those that were exposed without drug treatment. Since all the snails tolerated this relatively high dose of the inhibitor, we proceeded to examine whether pre -treating the susceptible snail with various doses of GA would affect their susceptibility phenotype.

As shown in Figure 6b, susceptible snails that were infected without prior drug treatment, as expected, were found to be shedding cercariae at 5 weeks PE (18.3%), with all untreated-exposed susceptible snails shedding cercariae at 9 weeks after infection. In contrast, susceptible snails that were treated with 100 mM GA prior to exposure failed to shed cercariae at the same 9 weeks PE time period while only a small percentage (3.7 to 4.8%) of snails pre-treated with lower doses of GA (0.1 to 10 mM) were found to be shedding cercariae. The longest surviving miracidial exposed, drug treated- (100 mM) snails remained negative (no cercarial shedding) for up to 9 months after infection. All snails not shedding cercariae by the 9^th^ week after exposure remained negative at week 12 and continued to be negative for at least 9 months PE.

The reduction of shedding (in percentages) from infected snails treated with the higher dose of GA compared to the lower doses was significant as determined by one-way ANOVA (*P*-value<0.05) [N = 60 and N = 36].

### GA treated miracidia remain infectious and develop successfully into the cercarial larval stage

**Figure 7:**
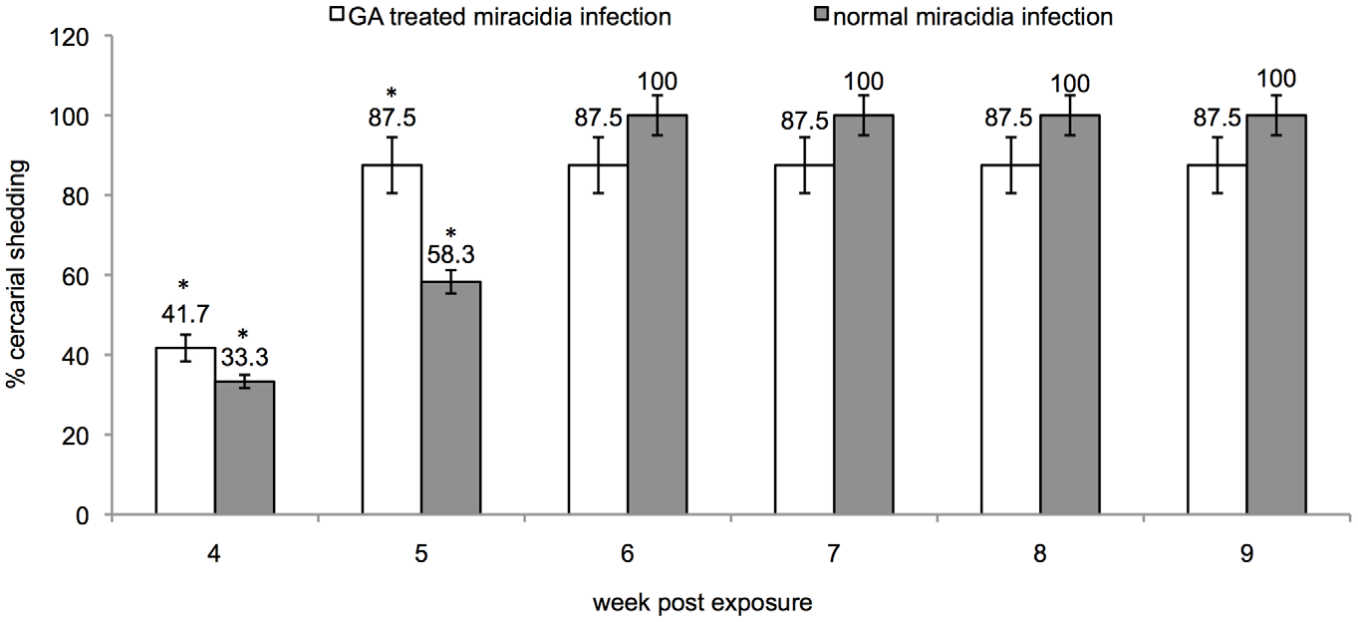
Cercarial shedding between normal, and GA-treated susceptible juvenile NMRI snails. Percentage cercarial shedding in NMRI snails infected with GA - treated miracidia (blank box) and normal miracidia (grey box). The snails were releasing cercariae after 4 weeks PE and continued to shed cercariae for the 9 weeks duration of the study. The asterisk* denotes a significant difference between GA-treated- and normal miracidia infection at weeks 4 and 5 PE. However note that after 6 weeks PE there is no significant difference between the two groups as determined by Student *t*-test with *P-value*<0.05 (error bar = SE; N = 60 in each group).

To rule out the possibility that results presented above might simply reflect the effect of the drug inhibiting the parasite's Hsp 90 homolog, thereby impairing the ability of miracidia to either penetrate the snail, or transform successfully into sporocysts, we treated miracidia directly with the highest dose of GA (100 mM) that was utilized in this study. These drug -treated miracidia were then utilized for snail exposures in comparison to exposures done with untreated miracidia, as control. In these experiments, more than 75% of miracidiae (with or without GA treatment) penetrated the snails within 5 min, and all (100%) successfully penetrated the snail within 1 hr (data not shown). These data were similar to penetration behavior we previously observed where we used either normal or irradiated miracidiae for snail exposures [22]. As shown in Figure 7, 41.7% and 87.5% of NMRI susceptible snails exposed to GA treated miracidia shed cercariae at 4–5 weeks. At 4 to 5 weeks PE, results analyzed by student's *t*-test showed that the percentage of cercarial shedding between NMRI snails exposed to either GA treated or normal miracidia was statistically significant (*P* –value<0.05). However, after week 5 PE, results showed that there was no statistically significant difference between the percentage of cercarial shedding between NMRI exposed to either GA-treated or normal miracidia. NMRI snails that were exposed to the untreated miracidia, likewise released cercariae after week 4 PE as we have routinely come to expect for snail infections performed by using this parasite strain and snail-host combination.

## Discussion

To date, very little information exists on molecular mechanisms that determine the outcome of the snail/schistosome interaction. Here, we have shown that upregulation of stress-related transcripts, such as those examined in this study, Hsp 70, Hsp 90 and RT in the *B. glabrata* snail host, soon after infection, plays an important role in their susceptibility to *S. mansoni*. These results are consistent with our previous data that showed a differential induction of stress genes occurs between juvenile susceptible and resistant *B. glabrata* snails after exposure to *S. mansoni* miracidia. Thus, upregulation of transcripts corresponding to Hsp 70 and RT was detected sooner, and more dramatically in susceptible compared to resistant snails following either heat shock or parasite infection [22]. Also, in this previous study, we showed that the stimulus/stimuli for this stress induction may be present in normal but not in irradiated attenuated miracidia.

Although we are yet to discover the nature of the parasite stress elicitor(s), it is most likely released from the incoming parasite as excretory secretory products (ESP). Interestingly, several studies have described using miracidial ESP to induce changes in either snail hemocytes, or the *B. glabrata* embryonic cell line, *Bge* [41]–[43]. Despite our limitation of not knowing what triggers the induction of stress in the snail (soon after exposure to miracidia) it is clear from this study that by using the heat -pulse regimen described to enhance the induction of stress to levels not typically seen under normal circumstances in resistant snails after exposure, it is possible to successfully reverse the resistant phenotype of juvenile resistant BS-90 snails i.e. render them susceptible to *S. mansoni*. Therefore, even though our results showed that parasite infection of the heat –pulsed snails caused a reduction in the induction of the stress transcripts, a more enhanced induction of all the three transcripts was still observed in heat-pulsed- infected snails than in snails that were exposed to miracidia alone without heat -pulse, helping to switch the phenotype of juvenile BS-90 snails from being resistant to susceptible. Thus, from these results, it is clear that resistance to *S. mansoni* in the juvenile BS-90 snail is a temperature-sensitive (*ts*) phenotype. Furthermore, cercariae released from these *ts* snails were infectious, developing fully into adult worms in the infected mouse, and with no change detected in DNA profiles of adult worms harvested from either these ‘resistant’ snails or our representative susceptible NMRI snail stock.

Historically, studies regarding *B. glabrata* susceptibility to *S. mansoni* have emphasized either the role of genetics, or innate defense in the snail/parasite association. While these areas of study have been pivotal in explaining some of the complex dynamics behind why schistosomes are either destroyed or survive in the snail host, it is clear from our results that temperature sensitivity of the stress gene loci reported is a contributing factor in the outcome of this host/pathogen interaction. Accordingly, we can only speculate that the *ts* resistant BS-90 snail's phenotype must involve changes in the activation of the three stress response genes to account for their altered kinetics. In a recent study, we showed that an unknown external stimulus/stimuli from the parasite was indeed able to mediate the non-random repositioning of gene loci of interphase chromosomes in the snail embryonic cell line, *Bge* [44]. These gene loci repositioning studies have since been reproduced in intact snails responding to *S. mansoni* (Arican, unpublished), and we are currently investigating if the stress genes are repositioned upon induction.

Previous studies have shown that the induction of stress is important for successful outcomes of other host-pathogen relationships as well. For example, in baculovirus infected Sf-9 cells, an increase in the expression of Hsp 70 was found to correlate with active virus replication. Thus, in this study, Lyupina et al. showed that inhibiting Hsp70 expression by the drug KNK437 suppressed virus replication [45]. Additionally, Hsp 90 has been shown to be essential for the growth of the malaria-causing agent, *P. falciparum*, in human erythrocytes [46]. Consequently, derivatives of GA, the Hsp 90 inhibitor used in the present study, has also been used to inhibit the growth of *P. falciparum* and another protozoan, *Trypanosoma evansi* [46], [47]. GA is a benzoquinone ansamycin antibiotic that binds to the N-terminal ATPase site of Hsp 90 to inhibit its chaperone activity. Although the inhibitor and its derivatives have previously been used to inhibit cell proliferation in cancer [48] and the growth of other parasites, as mentioned above it has never been shown, until now to treat any mollusk in the context of examining host-pathogen interactions. Interestingly, our results showed that GA treatment neither impaired the penetration behavior of the miracidia nor their ability to remain infectious. More studies, especially performed in *vivo* for longer time points will be needed to further examine this apparent lack of GA toxicity on the larval parasites. A similar lack of GA toxicity, which might be due to non-binding of GA to Hsp 90 homologues of free-living nematode larval stages, has previously been reported [49].

Heat shock proteins are highly conserved proteins that have been shown to play a critical role in maintaining protein integrity, preventing the aggregation of misfolded proteins in the cell, thereby maintaining normal cell function in the face of cellular injury from physical or physiological stress [50]. Very few studies have examined stress induction in relation to mollusk/pathogen interactions. Our results showing the very early (within 15 min) induction of Hsp 90 in susceptible snails (but not resistant snails) after infection was surprising and underscores the need for more studies on this stress protein, especially in relation to snail-schistosome interactions. Hsp 90 is expressed abundantly even in the absence of stress and constitutes a large portion of constitutively expressed protein in cells. The protein is regarded as being essential to cell viability because of its central role in forming complexes with a wide variety of co-chaperones and client proteins that are involved in major cellular pathways, such as signal transduction and cell-cycle control [51].

How Hsp 90 interacts directly, or indirectly with either the *B. glabrata* Hsp 70 or *nimbus* RT has yet to be investigated. Particularly, whether (or not) key molecules of the snail's innate defense system, such as FREPs are client proteins of Hsp 90, remain to be investigated. Previously, we showed that co-induction of Hsp 70 and *nimbus* RT transcripts occur soon after *S. mansoni* infection of juvenile susceptible snails [22]. Mobile Genetic Elements (MGEs), such as *nimbus* are responsive to cellular stress [52], [53]. However, the role of the *nimbus* non-LTR retrotransposon in the stress pathway *of B. glabrata* remains unknown. Further studies are, therefore, required to elucidate the relationship between all these stress genes (Hsp 90, Hsp 70 and RT) in the snail's behavior towards *S. mansoni*.

In other mollusks, such as the clam, *Mercenaria mercenaria*, the upregulation of Hsp 70 was observed in this mollusk in response to the opportunistic parasite, known as Quahog Parasite Unknown, QPX [54]. Also, in another clam, *Meretrix meretrix*, it has recently been shown that expression of Hsp 70 was upregulated soon after *Vibrio parahaemolyticus* infection [55]. Additionally, in the disk abalone, *Haliotis discus*, a recent molecular characterization showed that Hsp 90 is induced within 4 hrs after treatment with lipopolysaccharide, LPS [56]. In another *B. glabrata* susceptible snail, the M-line stock, Hanington et al. [26] showed upregulation of stress related transcripts following infection of these snails with trematodes, either *S. mansoni* or *Echinostoma paraensei*. The modulation (down regulation) of Hsp 70 in hemocytes isolated from exposed *B. glabrata* resistant and susceptible snails (the cells most intimately associated with the active destruction of schistosomes in the snail host) has also been reported, suggesting an involvement of this stress protein in the snail host's defense system [7], [23].

Indeed, an immunological role for stress proteins has been widely documented [57]. Thus, it might be reasonable to assume that in the snail/schistosome system, cellular stress triggered against parasite proteins that are recognized in the snail as non-self, by maintaining the homeostasis of the host, paradoxically protects the parasite as well. Larval schistosomes have been shown to express RNA transcripts for heat shock proteins. Presumably, such heat shock proteins (released from the parasite) might induce stress genes in the snail host, providing the cytoprotection that the parasite needs for its own successful invasion. While a strong anti-schistosome Hsp 70 humoral response has been reported in several infected (*S. mansoni* and *S. hematobium*) mammalian (murine, human and baboon) hosts [58], [59] nothing is known about the role of schistosome heat shock proteins and the snail's innate defense system. Thus far, we have evidence showing that an active defense system plays an important role in the BS-90 resistant snail's ability to ward off the parasite infection. By showing in this study that the deliberate use of stress in the form of non-lethal heat-pulse (boosting the level of inducible stress in the resistant BS-90 snail before infection) was a necessary step in rendering these normally robust resistant snails susceptible, we can suggest that the stress induced dampened the anti-schistosome response that is typically seen in these snails. Previously, it was shown that the resistance phenotype can be interfered with in resistant snails (10-R2 and 13–16-R1 stocks) if snails were first infected with other trematodes, such as *E. paraensei* and *E. lindoense* before being exposed to *S. mansoni* [60]. While no molecular explanation was given for this apparent suppression of the defense system by the dual infection protocol first described by Lie and Heyneman et al. [61], these early results showed that susceptibility to *S. mansoni* in these resistant stocks developed shortly (within 1 hr) after they had been exposed to *E. paraensei*. In light of our current results, we can assume that in this previous study, the primary echinostome infection, by triggering a stress response, dampening the innate defense system allowed the secondary *S. mansoni* infection to survive and develop. In other host-pathogen systems, there is clear evidence that expression of stress proteins, in particular Hsp 70 is an important feature in modulating the host innate immune response [62].

Another plausible explanation for the results presented here might be that the initial heat -pulse could have destroyed the resistant snail's hemocytes, thereby rendering these cells incapable of killing the incoming miracidia. It is also possible that the induction of stress might be a reflection of the successful establishment of the parasite in the snail. As mentioned above several factors, and not hemocytes alone govern compatibility/incompatibility issues between *B. glabrata* and *S. mansoni*. The heat pulse regimen utilized to enhance the induction of stress genes in the resistant snails was not lethal. All the snails survived at this elevated temperature, and a colony of BS-90 snails that we maintain (now in their eighth month) at 32°C continue to thrive. Interestingly, all (100%) of progeny snails (F1, exposed to miracidia and kept at 32°C after exposure) bred from BS-90 snails maintained at 32°C were found to shed cercariae at 3 to 4 weeks PE (Ittiprasert, Miller and Knight unpublished).

While we have no data supporting the notion that higher prevailing environmental temperatures might facilitate snail susceptibility, our results show that it is possible that climate change might impact resistant snail susceptibility to schistosomes. Indeed, a recent study showed that global warming might result in an increase in cercarial output of infected snails [63].

By using a recently developed gene silencing method based on soaking snails in siRNA coupled to the inert cationic carrier, polyethylene imines (PEI) [44], we are currently working to systematically knock-down transcription of Hsp 70, Hsp 90 and *nimbus* RT in the snail by the PEI delivery tool. Thus far, preliminary results indicate that knocking down these transcripts to levels comparable to those we routinely obtain for suppressing the expression of low copy RNA transcripts will be more challenging. Despite these initial challenges, we hope to elucidate the role of these stress proteins in the snail host schistosome relationship by knocking down their corresponding transcripts.

In addition, we have used the Hsp 90 inhibitor drug used in this study, GA, for treating pre-patent (2 week exposed snails) and results show consistently that once established, the drug has no effect on the infection and all treated pre-patent snails go on to shed cercariae.

In conclusion, we have shown in this study that by applying stress in the form of mild heat pulse to resistant BS-90 snails before they are exposed to *S. mansoni*, renders these snails susceptible. In contrast, infecting these snails before stressing them does not reverse their resistance phenotype, suggesting that the stress induction is an early necessary step in the sequence of molecular events that contribute towards making a snail susceptible. In addition, use of the stress inhibitor to treat susceptible snails before exposure was able to prevent them from shedding cercariae, again confirming that the stress pathway is indeed required for snail's to succumb to the parasite infection. These data open up a new opportunity to delve into unraveling the mechanism(s) that helps snails to either overcome or sustain the *S. mansoni* parasite infection, investigations that should lead to developing novel tools to interfere with schistosome-snail infections and thus reduce transmission of schistosomiasis.
